# High Cycle Stability of Hybridized Co(OH)_2_ Nanomaterial Structures Synthesized by the Water Bath Method as Anodes for Lithium-Ion Batteries

**DOI:** 10.3390/mi13020149

**Published:** 2022-01-19

**Authors:** Longlong Ren, Linhui Wang, Yufeng Qin, Qiang Li

**Affiliations:** 1 College of Mechanical and Electronic Engineering, Shandong Agricultural University, Taian 271018, China; renlonglong@sdau.edu.cn; 2 College of Information Science and Engineering, Shandong Agricultural University, Taian 271018, China; linhuiwang@sdau.edu.cn; 3 College of Physics, University-Industry Joint Center for Ocean Observation and Broadband Communication, Qingdao University, Qingdao 266071, China; liqiang@qdu.edu.cn

**Keywords:** Co(OH)_2_, structure hybridization, lithium-ion diffusion-controlled mechanism, anodes, lithium-ion batteries

## Abstract

Cobalt oxides have been intensely explored as anodes of lithium-ion batteries to resolve the intrinsic disadvantages of low electrical conductivity and volume change. However, as a precursor of preparing cobalt oxides, Co(OH)_2_ has rarely been investigated as the anode material of lithium-ion batteries, perhaps because of the complexity of hydroxides. Hybridized Co(OH)_2_ nanomaterial structures were synthesized by the water bath method and exhibited high electrochemical performance. The initial discharge and charge capacities were 1703.2 and 1262.9 mAh/g at 200 mA/g, respectively. The reversible capacity was 1050 mAh/g after 150 cycles. The reversible capability was 1015 mAh/g at 800 mA/g and increased to 1630 mAh/g when driven back to 100 mA/g. The electrochemical reaction kinetics study shows that the lithium-ion diffusion-controlled contribution is dominant in the energy storage mechanism. The superior electrochemical performance could result from the water bath method and the hybridization of nanosheets and nanoparticles structures. These hybridized Co(OH)_2_ nanomaterial structures with high electrochemical performance are promising anodes for lithium-ion batteries.

## 1. Introduction

To solve the problems of growing exhaustion of fossil energy (petroleum, natural gas, and coal) and the resulting environmental issues, many energy conversion and storage systems, such as lithium-ion batteries (LIBs), nanogenerators, and supercapacitors, have been extensively investigated [1,2,3,4,5,6,7,8,9,10,11,12,13]. LIBs have attracted much attention owing to their low self-discharge, no memory effect, high working voltage, and high energy density [14,15,16,17,18,19]. However, the specific capacity, power density, and rate capability of LIBs should be further improved to meet the demands of high-power energy storage systems [17,18,19]. One of the obstacles is the low theoretical capability of the commercial graphite anodes (372 mAh/g) [17,18,19]. According to the conversion reaction with lithium ions, transition metal oxides are promising anodes for LIBs due to their high theoretical capacities (500–1000 mAh/g) [18,19]. To achieve practical application, cobalt oxides, including CoO and Co_3_O_4_, have been intensely investigated to solve the intrinsic disadvantages of volume change and low electrical conductivity during the discharge-charge process [20,21,22,23]. As a precursor, Co(OH)_2_ has always been used to prepare cobalt-based oxides by heat treatment [24,25,26]. Ma et al. prepared CoO microsphere anodes for LIBs, which were evolved from the Co(OH)_2_ precursor in a high-temperature hydrothermal reaction [24]. Chen et al. reported the transformation from Co(OH)_2_ to Co_3_O_4_ nanosheets by annealing in air at 600 °C [25]. When used as anodes for LIBs, Co_3_O_4_ nanosheets exhibited a reversible capacity of 700 mAh/g. In addition, Co(OH)_2_ has also been used as part of nanocomposites to improve the electrochemical performance of the cobalt-based oxide anodes [27,28,29]. Huang et al. reported the improved electrochemical performance of Co(OH)_2_/Co_3_O_4_ nanocomposite anodes due to the introduction of Co(OH)_2_ and the resulting ordered nanostructures [27]. Li et al. prepared Co(OH)_2_/Co_3_O_4_/Co nanoparticle anodes, which showed a high reversible capacity of 540 mAh/g after 300 cycles with no obvious attenuation due to the hybridized effect of Co_3_O_4_ and Co(OH)_2_ [28]. However, investigations of the use of bare Co(OH)_2_ as anode for LIBs has rarely been reported, perhaps because of the complexity of hydroxides [30,31,32,33,34,35]. Through a simultaneous hydrothermal method, Ma et al. firstly prepared Co(OH)_2_-graphene nanosheet anodes, which exhibited an initial discharge capacity of 1599 mAh/g at 200 mA/g [30]. Wang et al. prepared α-Co(OH)_2_ with 3D flower-like morphology, which showed a high initial capacity of 1765 mAh/g [32]. Yoon et al. focused on the investigation of the exceptional reaction of Co(OH)_2_ and found a high initial capacity of 1122 mAh/g [33]. Recently, Shenouda et al. investigated the influence of composition ratios of Co(OH)_2_ and graphene on the electrochemical performance and found a reversible capacity of 690 mAh/g after 100 cycles [34]. Based on the above reports, even though high initial discharge capacities were observed, the cycle stability and the rate capability of the Co(OH)_2_ nanomaterials should be further enhanced to meet the demands of practical application.

In this work, we designed and prepared by the water bath method hybridized Co(OH)_2_ nanomaterial structures, which exhibit outstanding electrochemical performance as anodes for LIBs. The initial discharge and charge capacities were 1703.2 and 1262.9 mAh/g at 200 mA/g, respectively. The reversible capacity was 1050 mAh/g after 150 cycles, higher than the theoretical capacity (576 mAh/g) of Co(OH)_2_. The reversible capability was 1015 mAh/g at 800 mA/g and increased to 1630 mAh/g when returned back to 100 mA/g. The superior electrochemical performance could result from the water bath method used and the hybridization of nanosheet and nanoparticle structures. These hybridized Co(OH)_2_ nanomaterial structures with high electrochemical performance are promising anodes for lithium-ion batteries.

## 2. Experimental Section

### 2.1. Materials and Methods

**Figure 1:**
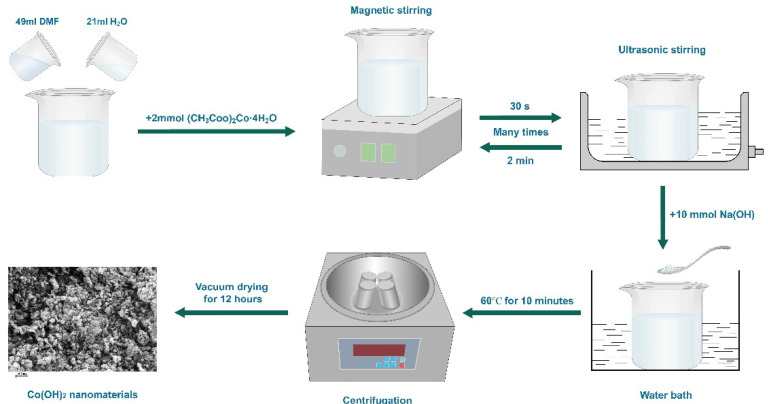
The illustration of preparing Co(OH)_2_ nanomaterials by the water bath method.

A schematic showing the preparation of Co(OH)_2_ nanomaterials is shown in Figure 1. The details of the preparation procedure are as follows: (CH_3_ COO)_2_Co·4H_2_O (2 mmol) was added to a mixed solution of pure water (21 mL) and dimethylformamide (DMF, 49 mL). After magnetic stirring (30 s) and ultrasonic stirring (2 min), in turn, several times, hexadecyltrimethyl ammonium bromide (CTAB, 8 mmol) was added. After ultrasonic stirring for another 30 min, NaOH (10 mmol) was subsequently added, and then magnetic stirring in a 60 °C water bath continued for 10 min. Finally, the Co(OH)_2_ nanomaterials were obtained after centrifuging with ethanol and pure water in turn and vacuum drying for 12 h at 60 °C. The assembly details of the half cells (CR-2032) were described before [17,18,19], and the main process is as follows: the Co(OH)_2_ nanomaterials, carbon black, and CMC (10 wt% in pure water) were mixed in a weight ratio of 7:2:1. After thoroughly grinding the mixture, the resulting black slurry was smeared on copper foil and then dried under vacuum at 60 °C overnight. The loading mass of active materials on the copper foil was about 0.71 mg/cm^2^. After punching the copper foil into many disks, the half cells were assembled in an argon-filled glove box with lithium metal foil as counter electrode. The diaphragm and electrolyte are a Celgard 2250 film and 1 M LiPF_6_ dissolved in a mixed solution of EC (50 *v*/*v*%) and DEC (50 *v*/*v*%).

### 2.2. Structure and Morphology Characterization

The nanomaterials’ structure was characterized by X-ray diffraction (XRD, Smart Lab, Rigaku, Tokyo, Japan) in the range of 5° to 80°. The morphology was further confirmed using a scanning electron microscope (SEM, Gemini SEM300, Zeiss, Oberkochen, Germany).

### 2.3. Electrochemical Performance Characterization

The electrochemical performance and impedance characteristics were tested on a battery testing system (Land-ct2001A, LanHe, Wuhan, China) and electrochemical workstation (CHI660E, ChenHua, Shanghai, China) at room temperature in the potential range of 0.01–3.0 V.

## 3. Results and Discussion

### 3.1. Structure and Morphology

**Figure 2:**
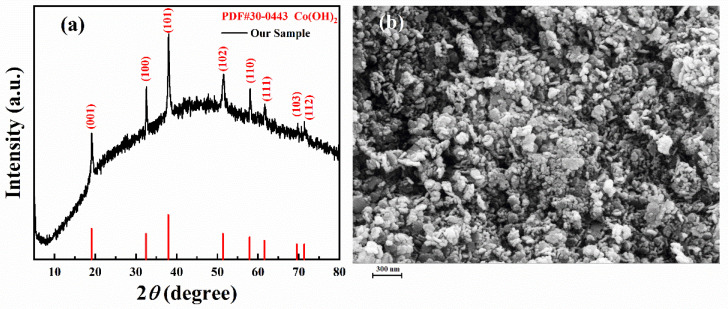
(**a**) XRD patterns and (**b**) the SEM image of Co(OH)_2_ nanomaterials.

The XRD patterns of the as-prepared precipitates are shown in Figure 2a. The diffraction peaks are in agreement with the standard cards of PDF No. 30–0443 (Co(OH)_2_). The characteristic peak at 19.1°, 32.5°, 37.9°, 51.4°, 57.9°, 61.5°, 69.8°, and 71.9° corresponds to the (001), (100), (101), (102), (110), (111), (103), and (112) crystal planes of the hexagonal Co(OH)_2_ phase, respectively [27,36]. There are no other diffraction peaks, which indicates the purity of the Co(OH)_2_ nanomaterials. From the SEM images shown in Figure 2b, the Co(OH)_2_ nanomaterials are composed of nanoparticles and nanosheets. The average diameter of the nanoparticles and the average thickness of the nanosheets are about 50 nm, while the length of the nanosheets cannot be seen clearly.

**Figure 3:**
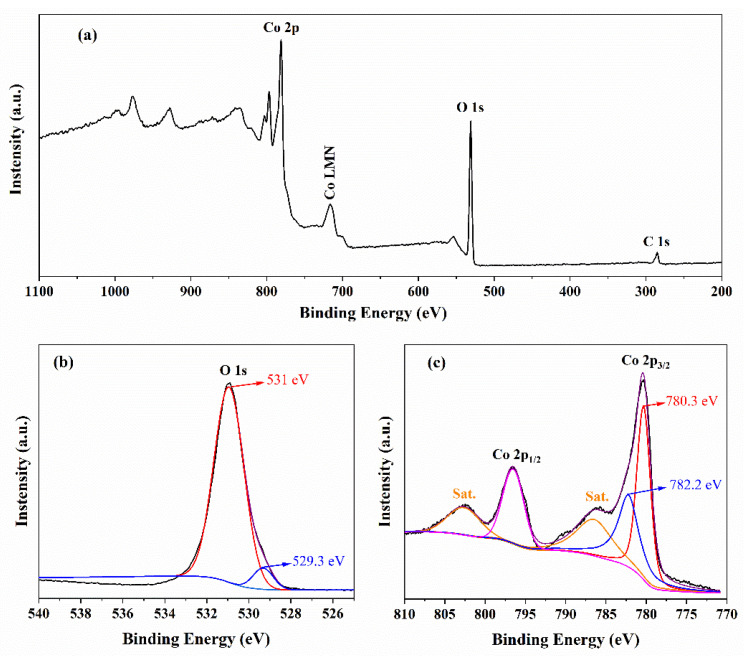
(**a**) The survey of XPS spectra of the Co(OH)_2_ nanomaterials. (**b**) the high resolution of O 1s peaks. (**c**) the magnification of Co 2p XPS spectra.

To further confirm the elements’ valence states in the Co(OH)_2_ nanomaterials, X-ray photoelectron spectroscopy (XPS) was performed, as shown in Figure 3. Figure 3a shows the survey XPS spectra of the Co(OH)_2_ nanomaterials, including C 1s, O 1s, and Co 2p peaks. From the high-resolution of O 1s peaks shown in Figure 3b, two fitted peaks at 529.3 eV and 531 eV were obtained, which would be consistent with the presence of H-O bonds and Co^2+^ binding to OH [33,35,36,37]. The Co 2p peaks were magnified, as shown in Figure 3c. There are two main peaks at 780.45 eV and 796.65 eV with a spin-energy separation of 16.2 eV corresponding to Co 2p_2/3_ and Co 2p_1/3_ of Co(OH)_2_, respectively [24,38]. There are also two satellite peaks located at 786.2 eV and 802.5 eV, which could be the satellite peaks of Co(OH)_2_ [36,37]. The two fitted peaks at 780.3 eV and 782.2 eV related to Co 2p_2/3_ further confirm that the as-prepared nanomaterials are Co(OH)_2_ [33,36,37,39,40].

### 3.2. Electrochemical Performance

**Figure 4:**
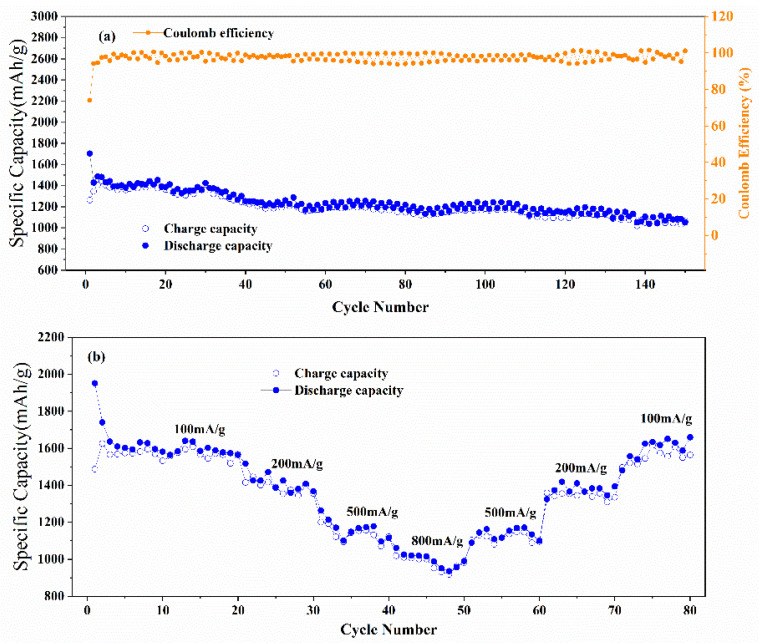
(**a**) Cycle performance and (**b**) rate capability of Co(OH)_2_ nanomaterials.

The Co(OH)_2_ nanomaterial electrodes exhibit high capacities, cycle stability at 200 mA/g, and outstanding rate capability, as is shown in Figure 4. Figure 4a shows the initial discharge and charge capacities were 1703.2 and 1262.9 mAh/g, respectively. The Coulombic efficiency increased to 94.21% in the second cycle and remained above 95% till the 150th cycle. A reversible capacity of 1050 mAh/g is obtained after 150 cycles, which is higher than the theoretical capacity (576 mAh/g) of Co(OH)_2_ [33,41]. The fluctuation of the capacities could result from the difference in testing temperatures during the day and night. However, the fluctuation does not influence the excellent cycle stability of the Co(OH)_2_ nanomaterials. The outstanding rate performance is shown in Figure 4b, The reversible capabilities of the Co(OH)_2_ nanomaterials were 1588, 1425, 1168, and 1015 mAh/g at 100, 200, 500, and 800 mA/g, and the capabilities increased to 1169, 1410, and 1630 mAh/g when the current density went back to 500, 200, and 100 mA/g. Table 1 compares the electrochemical results of other related Co(OH)_2_ materials and those prepared in our work, which indicates the outstanding electrochemical performance of the Co(OH)_2_ nanomaterials. The superior electrochemical performance could result from the facile water bath method and the structure hybridization of nanosheets and nanoparticles [42,43,44,45,46]. The nanoparticles filled in the nanosheets could avoid the aggregation of the nanosheets and then accommodate the volume change during the discharge-charge cycles [42,43,47].

**Figure 5:**
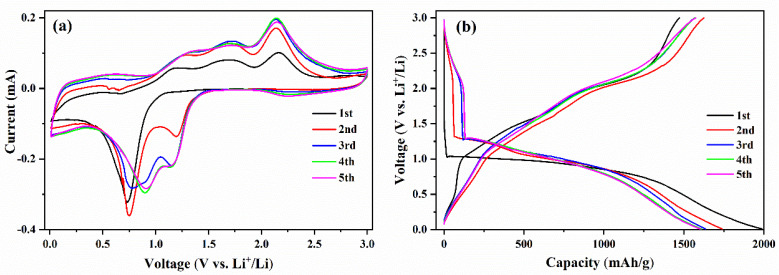
(**a**) The CV curves and (**b**) the discharge-charge curves of Co(OH)_2_ nanomaterials.

To clarify the electrochemical reactions of the Co(OH)_2_ nanomaterials, the first five cyclic voltammetry (CV) curves at 0.1mV/s were measured, as shown in Figure 5a. In the first cathodic sweep, there is only one broad peak at 0.72 V, which corresponds to the formation of the solid electrolyte interface (SEI) film and the reduction reaction of Co(OH)_2_ to Co [27,32,34,37,48]. For the first anodic process, there are three peaks at 1.20 V, 1.71 V, and 2.16 V, which corresponds to the multistep oxidation reaction of Co to Co(OH)_2_ [32,37,49]. The positions of the three oxidation peaks are almost unchanged in the following cycles, indicating the relatively stable reaction mechanism of the Co(OH)_2_ electrodes. In the second cathodic sweep, the main reduction peak at 0.72 V splits into two peaks at 0.75 V and 1.20 V, which has been reported to be due to the irreversibility of the Co(OH)_2_ structure after the first cycle or the size of the nanomaterials and nanoparticles of Co(OH)_2_ [32,38,49]. The two split peaks increase to high voltage a little in the third cathodic cycle, and then finally locate at 0.91 V and 1.45 V. After the first cycle, the reduction peak at 2.25 V appears and increases, which has also been reported in the study of Co(OH)_2_ nanosheets anodes [26]. This inconspicuous reduction peak could result from the insertion of lithium ions into the Co(OH)_2_ electrodes. The CV curves almost overlap after the third cycle, indicating the excellent stability of the electrochemical reaction during cycles [50,51].

The first five discharge-charge curves at 100 mA/g are shown in Figure 5b to compare with the CV curves shown in Figure 5a. In the first discharge curve, the voltage sharply dropped from the open-circuit voltage to 1.30 V, which was observed in many experimental results [26,27,28]. The voltage plateau from 1.04 to 0.64 V corresponds to the reduction reaction denoted by the peak of 0.72 V shown in Figure 5a. In the first charge curve, there are three voltage plateaus from 1.00 to 1.50 V, from 1.60 to 1.80 V, and from 1.95 to 2.47 V, which relate to the complex oxidation reaction to Co(OH)_2_ shown in CV curves. In the following cycles, there are two discharge voltage plateaus of 2.60–2.07 V and 1.34–0.75 V in the discharging process, relating to the lithium ions insertion denoted by the peak of 2.25 V and the multistep reduction reaction to Co denoted by the peaks of 0.91 V and 1.45 V in the CV cathodic process. The discharge-charge curves also almost coincide after the third cycle, indicating excellent cycle stability [29,30].

**Figure 6:**
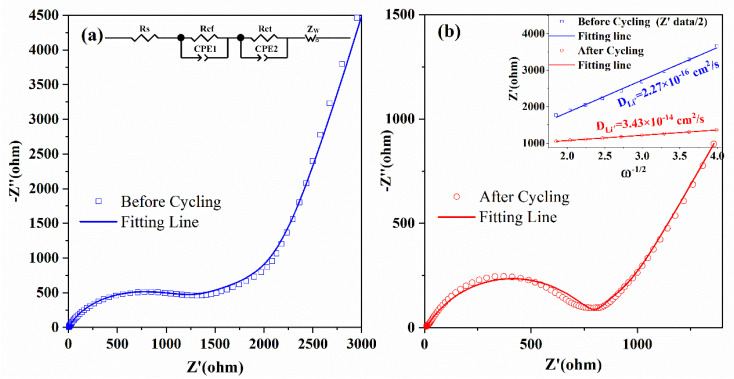
The EIS of Co(OH)_2_ nanomaterials before (**a**) and after (**b**) cycles. The inset in (**a**) is the equivalent circuit and the inset in (**b**) is the plots of *Z’* vs. *ω*^−0.5^.

The electrochemical reaction kinetic and the increased electrochemical performance of the electrodes can be investigated by electrochemical impedance spectroscopy (EIS) [17,18,19]. Therefore, the EIS of Co(OH)_2_ nanomaterials was measured from 10^−2^ to 10^5^ Hz before and after 50 cycles, as shown in Figure 6. The two Nyquist plots (scatters), which are composed of two semicircles in high frequency and a straight line in low frequency, can be well fitted by the equivalent circuit (fitting line) shown in the inset of Figure 6a. The parameters of *R*_s_, *R*_cf_, *R*_ct_, and *Z*_w_ denote the ohmic resistance of the electrode and electrolyte, the impedance of the SEI layer, the charge transfer resistance, and the Warburg impedance [17,18,19,50,52]. The Li-ions diffusion coefficient (*D*_Li^+^_) is an essential parameter of electrodes, and it can be obtained by the following equations:

(1) $$D_{{\mathrm{Li}}^{+}}=\frac{R^{2}T^{2}}{2A^{2}n^{4}F^{4}C^{2}\sigma^{2}}$$

(2) $$Z_{\mathrm{real}}=R_{s}+R_{\mathrm{ct}}+\sigma \omega^{-1/2}$$

The physical parameters above include the gas constant (*R*), the measuring temperature (*T*), the surface area of the electrode (*A*), the number of transferred electrons (*n*), the Faraday constant (*F*), the concentration of lithium ions (*C*), the Warburg coefficient (*σ*), and the angle frequency (*ω*), respectively [50,52]. The value of *σ* can be fitted by Equation (2) in the low-frequency [50,52]. As shown in the inset of Figure 6b, the impedance data before cycling was divided by 2, and *D*_Li^+^_ was obtained by Equation (1) further. All the fitted resistance parameters (*R*_s_, *R*_cf_, *R*_ct_, and *R*_total_) and *D*_Li^+^_ are shown in Table 2. Except *R*_s_ increased a little due to the formation of SEI film [18], the other resistances of (*R*_cf_, *R*_ct_, and *R*_total_) decreased remarkably after cycling, which indicates the increased electrochemical kinetics during cycling [17,18]. The Li-ion diffusion coefficient also dramatically increased after cycling, which is essential for the outstanding cycling performance and the rate capability [17,18]. The increased electrochemical kinetics and *D*_Li^+^_ of the Co(OH)_2_ nanomaterials could result from the hybridization of structures [42,43,44,45].

**Figure 7:**
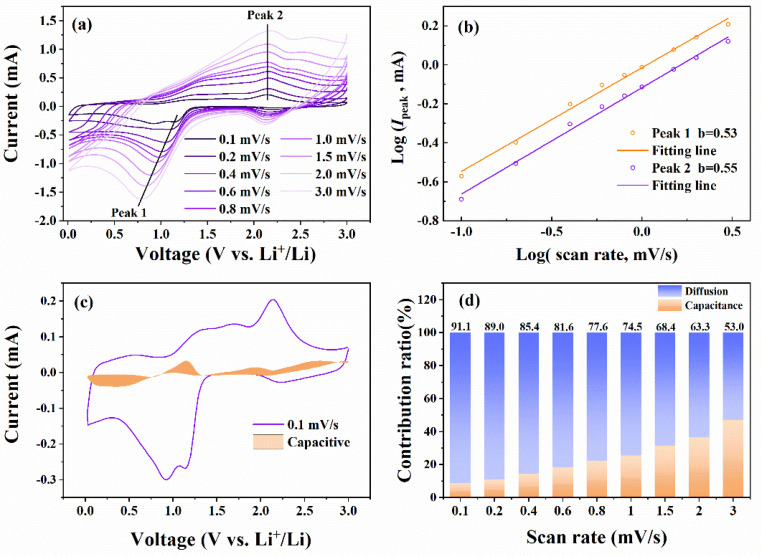
(**a**) The CV curves at different scan rates. (**b**) The corresponding plots of log(*I*_peak_) vs. log(*v*) at the two redox peaks marked in (**a**). (**c**) The CV curve and the contribution of surface capacitance mechanism at 0.1 mV/s. (**d**) Contribution ratios of diffusion-controlled mechanism.

The CV curves at different scan rates can investigate the lithium storage mechanism [22,44]. As shown in Figure 7a, the shapes of the CV curves are similar, indicating the stable electrochemical reaction mechanism. The Co(OH)_2_ nanomaterials show excellent lithium ion intercalation dynamics for the remarkable redox peaks at 3 mV/s [18]. The current in the CV curves was contributed by the lithium-ion diffusion mechanism and the surface capacitance mechanism, which the following equations can qualitatively obtained [18,19,22,43]:

(3) $$I_{\mathrm{peak}}=a\nu^{b}$$

(4) $$\log\left(I_{\mathrm{peak}}\right)=b\log(\nu )+\;\log a$$

where *I*_peak_ and *ν* denote the peak current in the CV curves and the scan rate. *a* and *b* are the adjustable parameters, which can be fitted by Equation (4) [22,43]. The value of *b* (0.5–1) can indicate the qualitative contribution rations of the lithium-ion diffusion mechanism and the surface capacitance mechanism [22,44,53]. For *b* = 0.5, the lithium-ion diffusion mechanism contributes almost the total current in CV curves, while for *b* = 1, the surface capacitance mechanism is dominant [22,44,53]. As shown in Figure 7b, the values of *b* for the two redox peaks marked in Figure 7a are 0.53 and 0.54, indicating the dominance of the lithium-ion diffusion contribution [18,19]. Because of the relatively high lithium-ion diffusion-controlled contribution at the two redox reactions, it is necessary to further quantitatively calculate the contribution ratios of the lithium-ion diffusion-controlled mechanism at different scan rates by the following equations [43,44,51,53]:

(5) $$I=k_{1}\nu +k_{2}\nu^{0.5}$$

(6) $$I/\nu^{0.5}=k_{1}\nu^{0.5}+k_{2}$$

where *k*_1_ν and *k*_2_*ν*^0.5^ denote the contribution of surface capacitance controlled and diffusion-controlled mechanisms, respectively [43,44]. The adjustable parameters *k*_1_ and *k*_2_ can be fitted by the linear fitting of Equation (6). After obtaining enough values of *k*_1_ and *k*_2_ at different voltages, the contribution ratios of surface capacitance controlled and diffusion-controlled mechanisms can be calculated [51,53]. As shown in Figure 7c, the contribution ratio of surface capacitance controlled is only 8.9%, while the diffusion-controlled mechanism contributes as high as 91.1% of the energy storage at 0.1 mV/s. Figure 7d shows the quantitative contribution ratios of the lithium-ion diffusion-controlled mechanism at different scan rates. Although the contribution of the lithium-ion diffusion-controlled mechanism decreases with the scan rates, it still is dominant in energy storage, which consists with the relatively low resistance and the high Li-ions diffusion coefficient obtained in EIS measurement [51,53].

**Figure 8:**
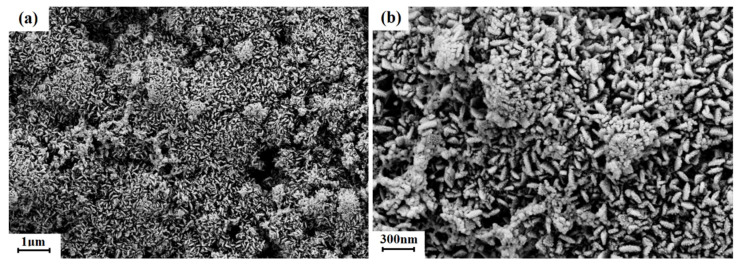
The SEM images of the Co(OH)_2_ nanomaterials after cycling tests with different magnification. (**a**) 1 μm, (**b**) 300 nm.

To investigate the structural stability of the Co(OH)_2_ nanomaterials during cycles, the electrodes were disassembled after the cycling test, and then the SEM images were recorded. As shown in Figure 8, the Co(OH)_2_ nanomaterials nearly maintain the nanosheet structure as before cycling, which is consistent with the good cycling stability and rate performance shown in Figure 4. The hybridization of structures could thus be a useful way to protect the structural integrity of Co(OH)_2_ nanomaterial anodes.

## 4. Conclusions

In this work, hybridized Co(OH)_2_ nanomaterial structures were synthesized by the water bath method and exhibited high electrochemical performance as anodes for LIBs. The initial discharge and charge capacities were 1703.2 and 1262.9 mAh/g at 200 mA/g, respectively. The reversible capacity was 1050 mAh/g after 150 cycles. The reversible capability was 1015 mAh/g at 800 mA/g and increased to 1630 mAh/g when cycled back to 100 mA/g. The superior electrochemical performance could result from the water bath method used and the hybridization of nanosheet and nanoparticle structures. The hybridization of structures could therefore be an efficient method to increase the electrochemical performance of Co(OH)_2_ nanomaterials as anodes for LIBs.

## Figures and Tables

**Table 1:** The electrochemical results of other related Co(OH)_2_ materials and our work.

Materials	Initial Discharge Capacity (mAh/g)	Reversible Capacity (mAh/g)	Current Density (mA/g)	References
Co(OH)_2_	1703.2	1050 (150 cycles)	200	This Work
Co(OH)_2_	1599.1	190.7 (20 cycles)	100	[25]
Co(OH)_2_	1232	614 (40 cycles)	100	[26]
GC–Co(OH)_2_	1146	706 (50 cycles)	58	[27]
Co(OH)_2_/Co_3_O_4_/Co@NGC	1032	543 (300 cycles)	100	[28]
ZnO@α-Co(OH)_2_	1425	1127 (150 cycles)	200	[29]
Co(OH)_2_@GNS	1599	910 (30 cycles)	200	[30]
α-Co(OH)_2_	1765	433 (50 cycles)	100	[32]
CS-Co(OH)_2_	1699.54	1036.32 (30 cycles)	0.1C	[33]
4Co(OH)_2_-1G	1250	690 (100 cycles)	0.1C	[34]
Mn–Co_2_(OH)_3_Cl	1966	1377 (50 cycles)	100	[35]
Co(OH)_2_–rGO	1410	690 (60 cycles)	50	[41]
Co(OH)_2_@MnO_2_	1621.33	700 (90 cycles)	250	[37]
Ni_x_Co_2x_(OH)_6x_@eRG	1308	787 (500 cycles)	200	[38]
Co_2_(OH)_3_Cl@GS	1600	753 (50 cycles)	200	[48]
Co(OH)_2_/GNSs	1654 (50 mA/g)	508 (100 cycles)	500	[49]

**Table 2:** The fitted parameters and *D*_Li^+^_ of Co(OH)_2_ nanomaterials.

States	*R*_s_ (Ω)	*R*_cf_ (Ω)	*R*_ct_ (Ω)	*R*_total_ (Ω)	$D_{{\mathrm{Li}}^{+}}({\mathrm{cm}}^{2}/s)$
Before cycling	3.29	1257	850.6	2110.89	9.10 × 10^−16^
After cycling	7.96	800.5	73.33	881.79	3.42 × 10^−14^
